# Surface Heterogeneity-Involved Estimation of Sample Size for Accuracy Assessment of Land Cover Product from Satellite Imagery

**DOI:** 10.3390/s19204430

**Published:** 2019-10-12

**Authors:** Huiqun Ren, Guoyin Cai, Mingyi Du

**Affiliations:** 1 School of Geomatics and Urban Spatial Informatics, Beijing University of Civil Engineering and Architecture, Beijing 100044, China; 2108521517043@stu.bucea.edu.cn (H.R.); dumingyi@bucea.edu.cn (M.D.); 2 Beijing Advanced Innovation Center for Future Urban Design, Beijing University of Civil Engineering and Architecture, Beijing 100044, China

**Keywords:** sample size estimation, land cover products, satellite images, surface heterogeneity, Jiangxi Province

## Abstract

Sample size estimation is a key issue for validating land cover products derived from satellite images. Based on the fact that present sample size estimation methods account for the characteristics of the Earth’s subsurface, this study developed a model for estimating sample size by considering the scale effect and surface heterogeneity. First, we introduced a watershed with different areas to indicate the scale effect on the sample size. Then, by employing an all-subsets regression feature selection method, three landscape indicators describing the aggregation and diversity of the land cover patches were selected (from 14 indicators) as the main factors for indicating the surface heterogeneity. Finally, we developed a multi-level linear model for sample size estimation using explanatory variables, including the estimated sample size (*n*) calculated from the traditional statistical model, size of the test region, and three landscape indicators. As reference data for developing this model, we employed a case study in the Jiangxi Province using a 30 m spatial resolution global land cover product (Globeland30) from 2010 as a classified map, and national 30 m land use/cover change (LUCC) data from 2010 in China. The results showed that the adjusted square coefficient of R^2^ is 0.79, indicating that the joint explanatory ability of all predictive variables in the model to the sample size is 79%. This means that the predictability of this model is at a good level. By comparing the sample size *N_S_* obtained by the developed multi-level linear model and *n* as calculated from the statistics model, we find that *N_S_* is much smaller than *n*, which mainly contributes to the concerns regarding surface heterogeneity in this study. The validity of the established model is tested and is proven as effective in the Anhui Province. This indicates that the estimated sample size from considering the scale effect and spatial heterogeneity in this study achieved the same accuracy as that calculated from a probability statistical model, while simultaneously saving more time, labour, and money in the accuracy assessment of a land cover dataset.

## 1. Introduction

Land cover provides basic geospatial information for applications in the fields of global environmental change, natural resources management, carbon and nitrogen cycle, and ecological monitoring [1,2,3]. Because of the continuous earth surface scanning and the correspondingly long-term data archives, satellite remote sensing is proven as an effective way in mapping global land cover and measuring land cover dynamic change [4]. Currently, national and international agencies have successfully created no less than ten global scale land cover datasets with spatial resolutions of 1 km, 500 m, 300 m, 30 m, and 12 m. These existing land cover datasets provide basic geographic information for studying climate, hydrology, environment, ecology, and urban regions [5,6,7,8]. Their accuracies are undoubtedly one of the most concerning issues for the potential users. Although data accuracy or uncertainty information is given at the same time as the land cover product releases [9,10], users in different application fields generally need to verify the accuracy before making a decision in using the land cover product [11,12].

Sampling inspection is a commonly used method for verifying the accuracy of land cover products. It provides reliable information on product quality and uncertainty [13,14]. Determining the sample size, as a key procedure in the sampling scheme, lays the foundation for the later stages of sample displacement and verification. A reasonably-estimated sample size is an effective way to avoid the phenomena of over-sampling or under-sampling [15,16]. In addition, the sample size also affects the number of investigators, and the cost and time of the field investigation. Therefore, the estimation of sample size is not only important theoretically, but also plays a guiding role in scientific research and field work investigation.

Currently, there are three major sample size estimation methods: empirical values, fixed-grid sampling, and calculation from a statistical model. The empirical values are the sample numbers required by researchers to test the accuracy of each category of classified satellite images. For example, Hay provided at least 50 empirical values for samples in each category initially [17], although the sample size could be enlarged with an increase in spatial regions and/or the amount of categories involved in the image classification. Congalton provided 75–100 sample data for each category of classified image, which are common empirical values for testing classified images [18]. Empirical sample sizes are simple and can achieve the goal of sample representation through spatial optimization in the process of sample placement. It is usually used to determine the sample size in remote sensing products, especially in an accuracy assessment of classified images. However, with the sprawl of spatial regions, the spatial heterogeneity can vary to a large extent around the entire region(s).

A fixed grid is another commonly used method for determining a sample size for performing an accuracy assessment. It divides the study area into regular grids with a certain size, for instance, 1 km or 10 km, and a sample from each grid is required [19,20]. Ridder used a 10 km × 10 km grid and randomly selected 9000 samples to assess the accuracy of a global forest dataset [21]. Stehman designed a 5 km × 5 km grid, in which 500 grids were randomly selected by synthesizing information on global climate zones and population density [22,23]. Through designing a sample encryption algorithm, a dataset for the verification of global land cover products was generated. This dataset can be used to validate other 1 km or 500 m resolution land cover datasets. The fixed grid sampling method is easy to implement, but the determination of grid specifications relies more on expert knowledge.

A statistical model is widely used to calculate a sample size. This method is based on theories of traditional probability and statistics [24,25]. Tong calculates sample sizes from two scales, one on the level of map divisions, and the other on the level of map elements [26]. By establishing probability distribution functions in each scale, the calculated sample size overcomes the problem of ‘too strict in map divisions and too wide in map elements’, which commonly exists in classical sampling schemes. Based on probability and statistics theory, Olofsson calculated a sample size for simple random sampling and stratified sampling methods respectively, using a statistical model [27]. As compared with the empirical or fixed-grid method, the statistical model is based on theories of statistics and probability. However, the sample size obtained by this method relies on the expected classification accuracy and sampling error information of the products. One of the main problems is that the same remote sensing product in different test sites with different areas will obtain the same numbers of samples, indicating that the surface spatial characteristics, such as patch numbers, aggregation, and diversity, from different study sites, are not involved in the calculation of sample size.

Until now, the determination of a sample size has primarily relied on expert knowledge or conditional assumptions. This often makes it difficult to ensure the rationality of the sample size. Therefore, this study addresses how to determine a number of samples while considering surface spatial heterogeneity.

Statistical theory is the foundation for determining the sample size. This study derived a sample size estimation using a stratified sampling approach. Then, a multi-level linear sample size estimation model was developed by considering the scale effect and surface spatial heterogeneity, with emphasis on two aspects of these issues. First, a watershed unit with ecological and geographical significance was introduced in this study as the basic spatial unit for performing the accuracy assessment, avoiding the objectivity issues existing in current spatial units of pixels or polygons [28]. Second, landscape indicators were employed to describe the surface heterogeneity and complexity. As the characteristics of the spatial heterogeneity would inevitably affect the sample size used for validating the land cover dataset, this study computed several major landscape indicators and assessed their impacts on the surface heterogeneity in watershed units, thereby reaching the goal of this study (to develop a reasonable model to estimate the sample size).

The remainder of the paper is organised as follows. Section 2 introduces the study area, data sources, and data pre-processing methods. Section 3 describes the commonly used statistical model of sample size estimation, the selection of scale factor and landscape indicators, and the development of the multi-level linear regression model. Section 4 presents results and an analysis of the developed model, and Section 5 provides preliminary conclusions.

## 2. Study Area and Data

### 2.1. Study Area

**Figure 1:**
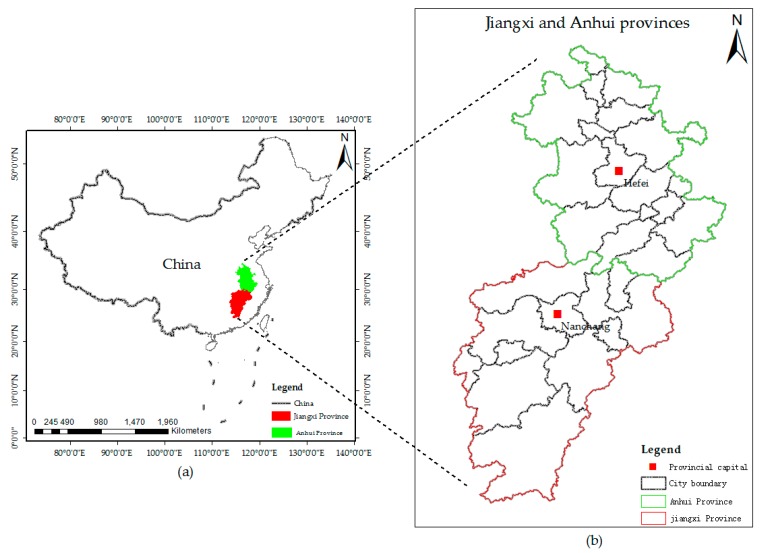
The study site (**a**) locations in China of (**b**) the Jiangxi Province and the Anhui Province.

The Jiangxi Province is located in south-eastern China, with a total area of 169,900 square kilometres. It belongs to a subtropical humid climate with abundant rainfall. The landform is dominated by mountains (36%) and hills (42%). The main land use types are forest lands and crop lands, with the forest coverage rate reaching 60%, ranking as first in China. The typical geomorphological and climatic features cause Jiangxi to be covered by various types of land covers. Therefore, we selected the Jiangxi Province as the study site to develop the sample size estimation model [29,30]. The Anhui province which is adjacent to the Jiangxi Province is selected to testify the developed model (Figure 1).

### 2.2. Data Sources

In this study, the global land cover dataset ‘Globeland30’ from 2010 was selected as the test dataset (http://www.globeland30.org). Globeland30 is a high-resolution land cover mapping product developed by the National Geomatics Centre of China, with a spatial resolution of 30 m. It has attracted the attention of researchers at home and abroad. The Globeland30 dataset includes 10 first-level classes. An approach based on the integration of pixel- and object-based methods with knowledge (POK) was used to extract land categories, effectively improving the classification accuracy [31].

The reference data is the national land use/cover change (LUCC) dataset from 2010 with a scale of 1:100,000, which was provided by the Institute of Geographic Sciences and Natural Resources Research, Chinese Academy of Sciences (CAS). The data was produced mainly by visual interpretation from remote sensing experts in China and was updated every five years. The LUCC data has six first-level classes and 25 second-level classes. Field investigation has shown that the LUCC classification accuracy is very high, with more than 90% overall accuracy (OA) for the second-level class. Both of these two datasets use the same major data sources of Landsat imagery [32,33]. In addition, the digital elevation model (DEM) dataset of the ‘Advanced Spaceborne Thermal Emission and Reflection Radiometer Global Digital Elevation Model’ (ASTGTM) (http://datamirror.csdb.cn) in the study area was downloaded to perform the division of the watershed unit.

### 2.3. Data Processing

#### 2.3.1. Classification System Transformation

**Figure 2:**
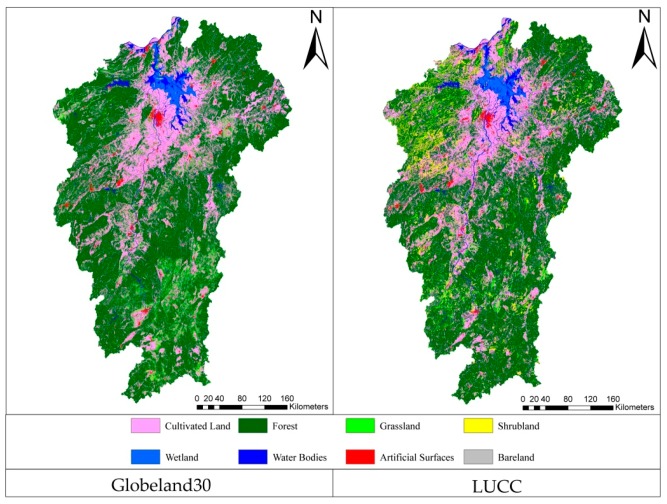
Globeland30 dataset and the reclassified land cover from land use/cover change (LUCC).

The Globeland30 and LUCC datasets must be pre-processed before accuracy assessment, owing to their differences in projection and classification systems. For avoiding area deformation, the Globeland30 dataset was projected to the Albert equal area projection (European Petroleum Survey Group (EPSG) code: 3857), which is the projection system employed for the LUCC dataset. Then, these two products were unified in the classification system. Based on the LUCC definition for each product, the classification transformation is presented in Table 1 [34,35]. Finally, we obtained the test and reference data, as shown in Figure 2.

#### 2.3.2. Digital Elevation Model (DEM) Processing

**Figure 3:**
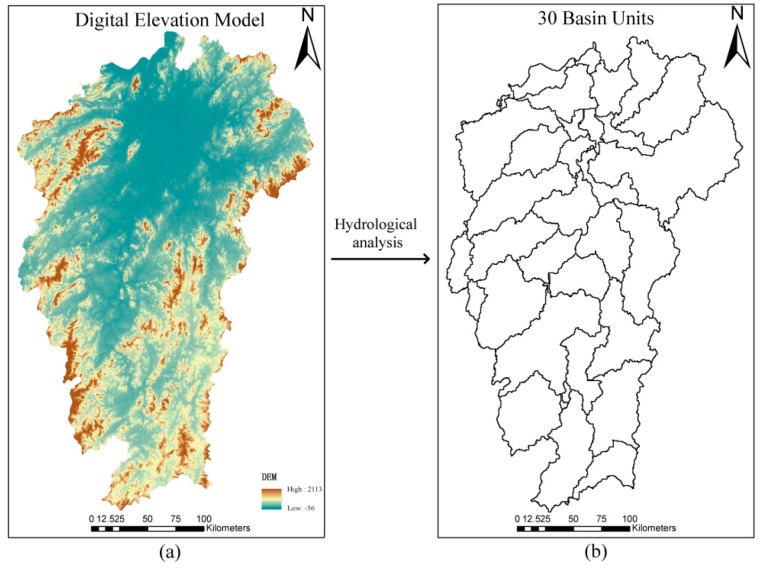
(**a**) 30 m ‘ASTER Global Digital Elevation Model’ (ASTGTM) digital elevation model (DEM) data and (**b**) the watershed units (calculated from a).

The DEM was projected into the Albert equal area projection system. A hydrological analysis was implemented by using ArcGIS 10.2 to calculate the watershed units. The watershed unit number was decided by the threshold value of the catchment surface [36,37,38]. After repeated experiments and visualization on the division of the watershed unit, we used a threshold value of flow accumulation of 4,000,000 for the catchment surface to obtain 48 basin units. Some units with small areas emerged adjacent to the unit that has the largest area. Finally, we collected 30 basin units as shown in Figure 3b, to perform the following study.

## 3. Methodology

### 3.1. Sample Size Determination from Probability Statistical Model

The estimation of the sample size is dependent on the sampling design method. For a land cover dataset with multiple categories, a stratified random sampling design has often been recommended in related research [39,40,41]. Cochran provided a set of rigorous calculation equations to obtain a sample size from the perspective of statistical theory, which is the foundation of latter research on sampling techniques [27,42,43]. According to the good practices for estimating accuracy recommended by Olofsson et al. [27], the commonly used equation for calculating sample size is:

(1) $$n=\frac{(\sum W_{h}S_{h}{)}^{2}}{V({\bar{y}}_{st})+(1/N)\sum W_{h}{S_{h}}^{2}}$$

where, *V*(${\bar{y}}_{st}$) is the standard error of the estimated OA, and is generally designated as 0.01, $W_{h}$ is the stratum weight (proportion of area of class i in the map), *S_h_ =* $\sqrt{p_{i}\left(1-p_{i}\right)}\;$ [43], $S_{h}$ represents the level *i* standard deviation, and $p_{i}$ is the user accuracy, which can be obtained through experiments [42,44].

### 3.2. Determination of Variables in a Multi-Level Linear Model

On the basis of the estimated sample size from the statistical model, this study attempted to develop a sample size estimation model by considering the scale effects of spatial units and the spatial heterogeneity reflected from the land cover product. As at least three factors, i.e., sample size from the statistical model, spatial effect, and heterogeneity characteristics, impacted the number of samples, this study employed a multi-level linear regression method to develop the sample size estimation, expressed as follows:

(2) $$N_{S}=A_{0}+nA_{1}+CA_{2}+LA_{3}=A_{0}+\frac{(\sum W_{h}S_{h}{)}^{2}}{V({\bar{y}}_{st})+(1/N)\sum W_{h}{S_{h}}^{2}}A_{1}+CA_{2}+LA_{3}$$

In the above, *N_S_* is the estimated sample size, *n* is the sample size calculated by the probability sampling method (Equation (1)), and C represents the sampling constraint on each watershed unit. Theoretically, the larger the test region is, the more samples are needed, and vice versa. In that regard, the ‘best’ sample constraints for regions with different areas should be discussed in this study. *L* is a set of spatial heterogeneity factors, including a number of landscape indicators that can indicate information on the fragmentation, diversity, and stability of the watershed units. *A*_0_, *A*_1_, *A*_2_, and *A*_3_ are regression coefficients, and will be discussed in Section 4.

**Figure 4:**
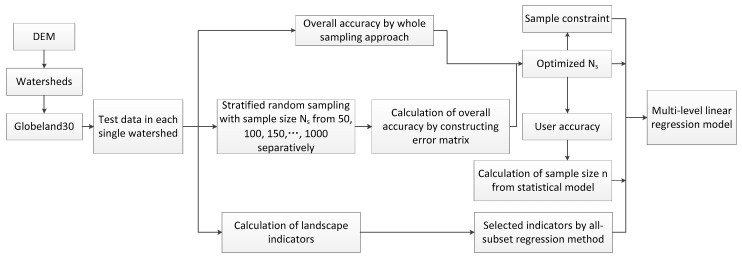
Procedures of parameter determination used in a multi-level linear regression method.

With the help of watershed units, we obtained Globeland30 and LUCC data as test and reference data respectively, for each single watershed. This following part mainly describes the calculation and determination of *n*, *N_S_*, *C*, *L*, and the other variables needed in Equation (2). The main procedures are shown in Figure 4.

#### 3.2.1. Determining *N_S_*, *n*, and *C*

**Figure 5:**
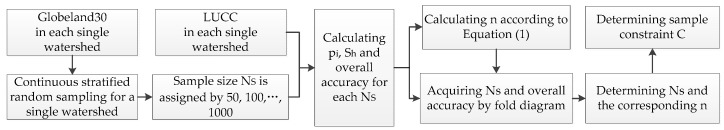
Flowchart of determining *N_S_*, *n*, and *C*.

Equation (1) in Section 3.1 is used to calculate the sample size *n*. We can see that all parameters in Equation (1) are available except for $S_{h}$. To ensure the optimization and specificity of the *n* value in each watershed unit, $S_{h}$ is determined by stratified random sampling for the land cover products of Globeland30. The main steps are as follows (Figure 5): First, each basin unit in the study area is sampled by the stratified random sampling method. Sample *N_S_* values are assigned by 50, 100, 150, …, 1000, i.e., with an interval of 50. Second, a user’s accuracy p_i_ from different samples *N_S_* in each basin unit is calculated from the constructed error matrix, while using LUCC as a reference data. Third, $S_{h}$ is obtained for each single basin unit according to the evaluated results.

As an example, Table 2 describes the $S_{h}$ results for the Number 2 basin unit. The value of *h* ranges from 1 to 8, representing eight different strata or land types of Globeland30. $W_{h}$ is the area weight information of each stratum in the basin unit. The $S_{h}$ for each basin unit can be obtained by the above-mentioned stratified random sampling.

After obtaining the parameter $S_{h}$, the sample size n of each *N_S_* can be calculated from Equation (1). As there are 10 different values of *N_S_* in each basin unit, there are 10 different corresponding values of *n*. The determination of *n* used in the simulation of the multi-level linear sample size model for a single basin unit should be based on the determination of *N_S_*.

**Figure 6:**
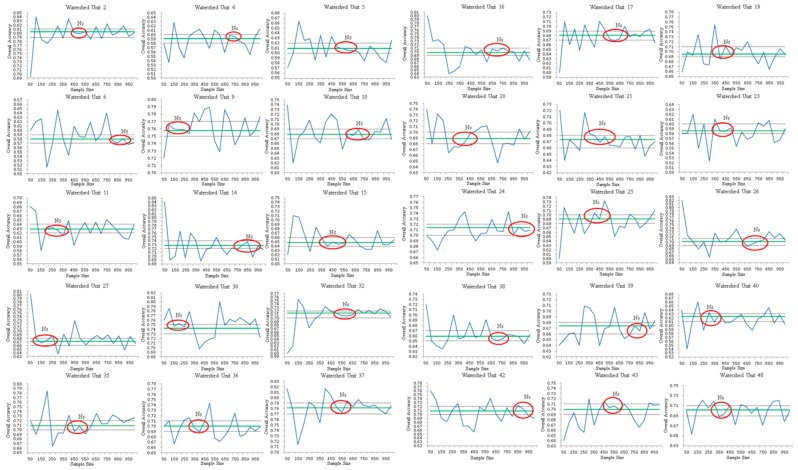
Determination of sample size *Ns*.

Figure 6 shows broken-line maps between the *N_S_* values obtained from the successive stratified random sampling of 30 basin units and the OA information. The blue line is the result from the stratified random sampling method, the green line represents the result of the accuracy assessed by the whole sample, the black dotted line gives the allowable precision range under the premise of an absolute error of 0.01, and the red circled part is the selected region where the OA varies with *N_S_* in the acceptable precision range. The first *N_S_* value in the circled region is regarded as the estimated sample size [45], aiming to obtain a reasonable result in accuracy assessment by using as few samples as possible. The sample size *n* corresponds to $N_{S}$. Once *N_S_* is determined, *n* can also be obtained. Finally, we obtained 30 records of *N_S_* and *n* from the 30 basin units.

Sample constraint *C* describes how large of a region can allocate a sample, which is a factor affecting the number of samples. In this study, the sample constraints of each basin unit were obtained by the following expression:

*C* = *N_S_/S* (3)

Here, *S* is the basin unit area (km^2^).

#### 3.2.2. Selection of Landscape Indicators

The landscape index, an index for quantitative analysis of landscape patterns, can measure the type, quantity, shape, spatial distribution, and complexity of the analysis units [46,47]. In recent years, an increasing number of studies have used the landscape index to describe spatial heterogeneity information, although their focus is not on the estimation of sample size, but on the layout of sample points [45,48] or land cover extraction [49,50]. According to the target of estimating the sample size of the surface coverage data, 14 landscape indicators were selected to describe the spatial heterogeneity information of the landscape levels in the watershed units from seven categories: area metrics, contrast metrics, edge metrics, shape metrics, proximity metrics, aggregation metrics, and diversity indexes. The ecological significance and descriptions of these indicators are presented in Table 3.

**Figure 7:**
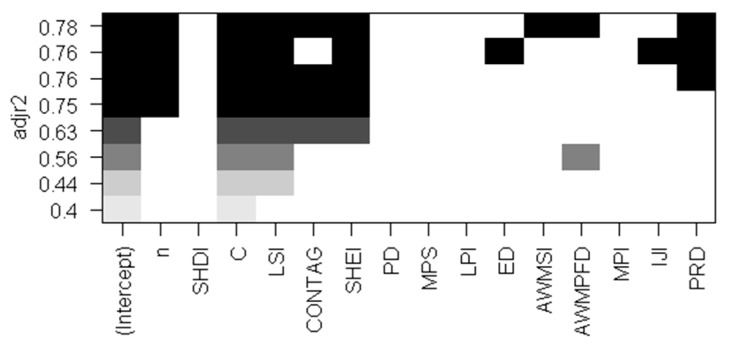
Result of all-subsets regression method.

We need to identify the most representative indicators from the 14 landscape indexes in the 7 categories. All-subsets regression, a commonly used method for feature selection, was employed to select the indicators. By adjusting the values of R^2^, the ‘best’ model was selected to determine the variables of the fitting model. As shown in Figure 7, we found that the adjusted R^2^ value of the ‘best’ model was 0.78, and the corresponding landscape indices were the landscape shape index (LSI), contagion index (CONTAG), Shannon’s evenness index (SHEI), area-weighted mean shape index (AWMSI), area-weighted mean patch fractal dimension (AWMPFD), and patch richness density (PRD). In addition, the R^2^ value of the sample size from the probability sampling theory and sample constraints in the fitting model is greater than 0.7, indicating that both of them are independent in the model for sample size estimation. Therefore, the 6 indicators of LSI, CONTAG, SHEI, AWMSI, AWMPFD, and PRD were selected for the following multi-level regression analysis.

#### 3.2.3. Regression Analysis

Equation (2) indicates that the sample size estimation is supposed to be a multi-level linear function of sample size from probability sampling theory, sample constraints, and spatial heterogeneity. Among them, coefficients *A*_0_, *A*_1_, *A*_2_, and *A*_3_ are determined by regression analysis. In this study, an ordinary least squares (OLS) regression model was used to determine these regression coefficients [51,52]. OLS is one of the most commonly used core methods in multi-level linear regression models. Its form is as follows:

(4) $${\hat{Y}}_{i}={\hat{B}}_{0}+{\hat{B}}_{1}X_{1i}+\cdots \cdots +{\hat{B}}_{k}X_{ki}$$

where, *i* = 1, 2, …, *n*, ${\hat{Y}}_{i}$ is the predicted value of the dependent variable corresponding to the *i^th^* observation, *X_ki_* denotes the value of the *j^th^* predictive variable corresponding to the *i^th^* observation, and ${\hat{B}}_{0}$ denotes the intercept term, i.e., the predicted value of *Y* when all of the predicted variables are zero. ${\hat{B}}_{k}$ is the regression coefficient of the predictive variable *j*, i.e., the change of Y caused by *X_j_* changing a unit.

The OLS model obtains regression coefficients by reducing the difference between the real values of response variables and the predicted values, i.e., by minimizing the sum of squares of residual errors:

(5) $$\sum_{i=1}^{n}{\left(Y_{i}-{\hat{Y}}_{i}\right)}^{2}=\sum_{i=1}^{n}{\left(Y_{i}-({\hat{B}}_{0}+{\hat{B}}_{1}X_{1i}+\cdots \cdots +{\hat{B}}_{k}X_{ki})\right)}^{2}=\sum_{i=1}^{n}{\varepsilon_{i}}^{2}$$

## 4. Result and Analysis

### 4.1. Multi-Level Regression

**Figure 8:**
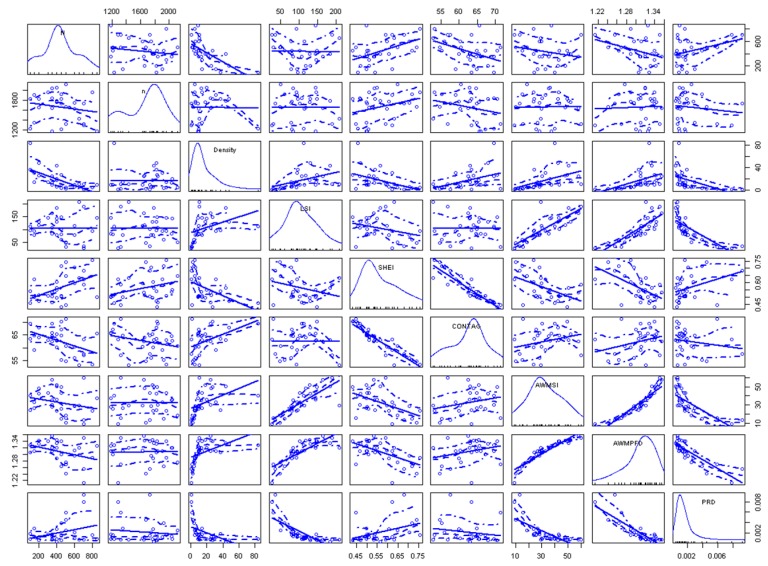
Scatterplot matrix between sample size *N_S_* and the predictive variables.

As shown in Figure 3b, there are 30 basin units in the study area. Therefore, we obtained a total of 30 records with optimised sample size *N_S_*. To show the correlations between sample size *N_S_* and the explanatory variables, a scatterplot matrix was obtained, and is presented in Figure 8. The diagonal area is the density map of the variables, whereas the blue line in the non-diagonal area represents the linear and smooth-fitting curves. From the matrix, every predictive variable has a tilt trend to some extent. The sample size *N_S_* decreases with the increase of sample constraints (*C*), SHEI, and AWMPFD, whereas it increases with an increase of CONTAG and PRD. This means that the relationship between the sample size *N_S_* and the independent variables is not a phenomenon of simple positive or negative correspondence.

After analysing the scatterplot matrix, the multi-level linear regression analysis was implemented, and the coefficients are shown in Table 4.

The column for Pr(>t) shows the significance of the regression coefficients of the independent variables, and the column of ‘Significance codes’ represents the degree of significance. The regression coefficients of AWMSI, AWMPFD, and PRD are not significant enough to pass the *t* test, and the multiple R^2^ of the model is different from the adjusted R^2^. This reflects a problem of instability. Therefore, the model needs to be improved. From additional experiments, we found that the regression coefficients of the independent variables are more significant when AWMSI, AWMPFD, and PRD are removed. Although the R^2^ value decreases, the stability performance improves (Table 5). As a result, only 3 indicators were involved in the final estimation of sample size.

Therefore, the multi-level linear regression equation can be expressed as follows:

(6) $$N_{S}=-7159-0.255n-9.261C+4.21LSI+77.9CONTAG+5085SHEI$$

By substituting Equation (1) into Equation (6), we finally obtain the estimation of the sample size by considering the scale effect and spatial heterogeneity characteristics, and it is expressed as follows:

(7) $$N_{S}=-7159-0.255\frac{(\sum W_{h}S_{h}{)}^{2}}{V({\bar{y}}_{st})+(1/N)\sum W_{h}{S_{h}}^{2}}-9.261C+4.21LSI+77.9CONTAG+5085SHEI$$

This relationship shows that LSI, CONTAG, and SHEI contribute positively to the sample size *N_S_*, whereas the sample size *n* of probability sampling theory and the sample constraint *C* contribute negatively to the sample size *N_S_*. CONTAG and SHEI contribute the most and play a vital role in the change of sample size. All of the predictive variables explain the variance of 79% of the sample size *N_S_*.

### 4.2. Model Verification

This study used a cross-validation method to test the generalization ability of the established OLS regression model. Cross-validation, as a commonly used model validation technology, has the advantage of high prediction accuracy [53]. Considering the small amount of data used to fit the model, three-fold cross-validation was used. Three-fold cross-validation was used to divide the original sample into three equal-sized sub-samples, one of which was retained as test data for model validation, while the other two sub-samples were used as training data. This process was repeated three times, and each of the three sub-samples was used as the validation data. The average R^2^ of the three cross-validation results was taken as the final estimation solution, as shown in Table 6. The results show that there are some differences between the original R^2^ and the three-fold cross-validation R^2^. The R^2^ value changes greatly after cross-validation, indicating that the stability between the variables and the generalization ability of the models is less than satisfactory.

**Figure 9:**
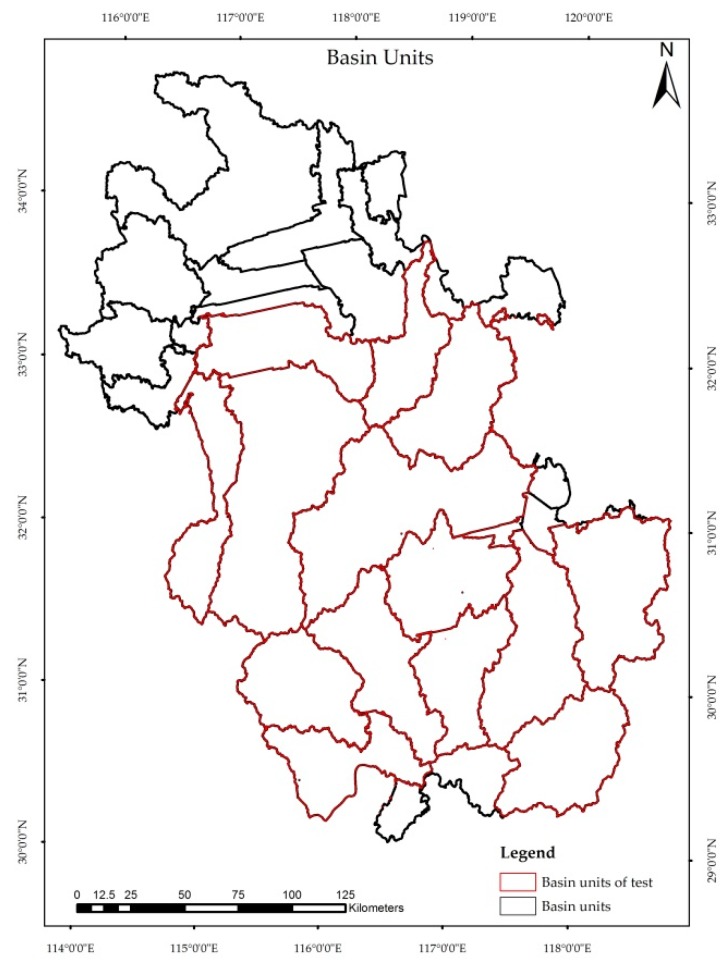
The divided watershed units in the Anhui Province.

In addition to the cross-validation, we applied the method of sample size estimation to the Anhui Province to test the developed model. Using the same method as mentioned above, we divided the Anhui province into 32 watersheds and selected 14 units to perform the model validation (Figure 9). The same datasets as those used in the Jiangxi Province were used to test this model. First, we calculated the sample size using a statistical model, i.e., Equation (1), and the developed model in this study, i.e., Equation (7), respectively. Then, we assessed the accuracy of Globeland30 by using the two above-calculated sample sizes. Finally, we compared the accuracies and the results are presented in Table 7. We can see that the difference of OA obtained from two different approaches is very small in most watersheds, the maximum and minimum difference is 4% and 0.1% respectively, with an average of 1.21%. In contrast to the similar accuracy, the sample sizes calculated by the developed model are smaller than that from the statistical model. For the employed 14 watersheds in the Anhui Province, there are 24,425 sample points calculated by the statistical model, while it is 12,399 computed by the developed model in this work. Compared with the statistical model, our developed model decreases the sample size by 49%. This indicates that a smaller sample size can achieve the same performance as the statistical model by considering the scale effect and surface heterogeneity.

### 4.3. Relative Importance of Predictor Variables

**Figure 10:**
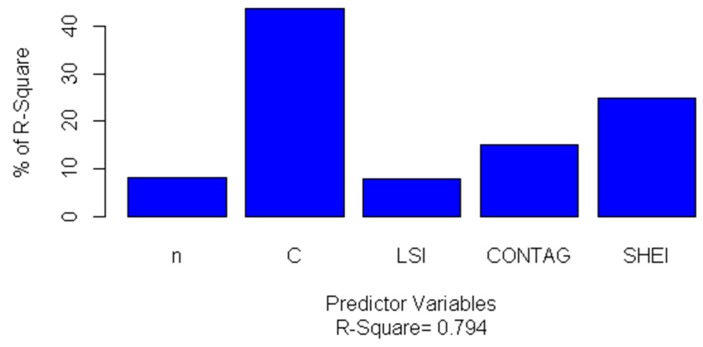
Relative importance of the predictor variables used in this study.

The OLS regression method is used to analyse the influence of the sample size *n* of the probability sampling theory, the sample constraints *C*, and spatial heterogeneity factors on the estimated sample size *N_S_*, and the relationship between them is obtained in Equation (7). The standardised regression coefficient method and the relative weight method are employed to evaluate the relative importance of the predictor variables in the multi-level regression analysis. The results are shown in Table 8 and Figure 10.

The standardised regression coefficient is the simplest method for predicting the relative importance of variables. It represents the expected change of response variables caused by the change of one standard deviation of a predictor variable when other predictor variables remain unchanged. Table 8 shows that when the other variables remain unchanged, SHEI changes by a standard deviation, and that the sample size will increase by 2.13 standard deviations, i.e., the most important relative to the sample size *N_S_*. In contrast, the sample size *n* of the probability sampling theory has the least relative importance to the sample size.

As compared with the standardized regression coefficient, the relative weight is a more promising method for predicting the relative importance of factors [54]. It ranks variables according to their contribution to R^2^. Figure 10 shows the relative importance of each factor. The results show that the sample constraint *C* accounts for 44% of R^2^, which is of the greatest relative importance to the sample size *N_S_*. It shows that the regional scale cannot be neglected in the sample size determination. SHEI and CONTAG, as spatial heterogeneity factors describing the aggregation and diversity of watershed units, explain 25% and 15% of R^2^, respectively. The remaining factors are LSI and *n*. Therefore, in terms of relative importance, the regional scale effect and spatial heterogeneity have an influence on the determination of the sample size in accuracy assessment of remotely sensed land cover products.

### 4.4. Analysis

**Figure 11:**
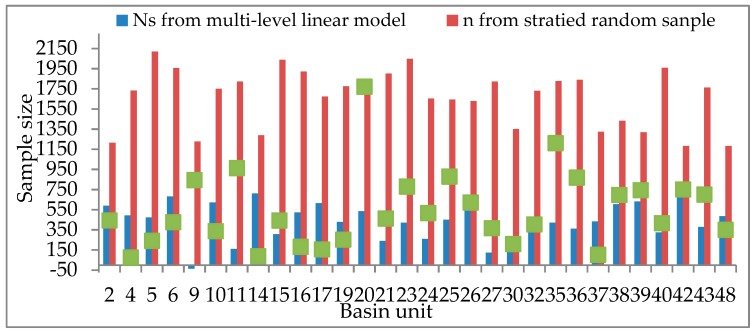
Histogram of sample sizes *n* and *N_S_*.

**Figure 12:**
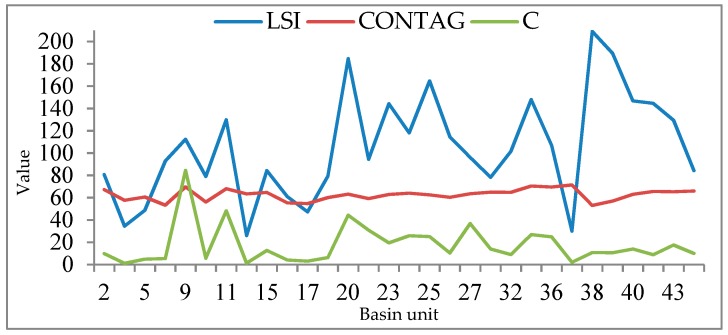
Diagrams of landscape shape index (LSI), contagion index (CONTAG), and *C*.

**Figure 13:**
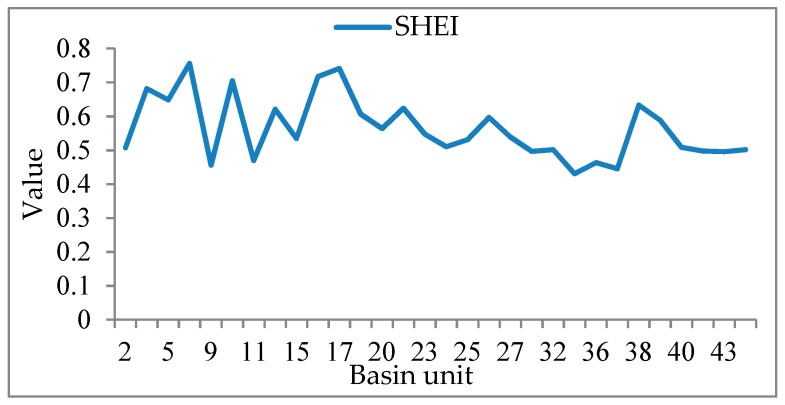
Diagram of Shannon’s evenness index (SHEI).

Figure 11 is a histogram of the sample size *N_S_* obtained from the multi-level linear model and sample size *n* and based on probability and statistics theory. Figure 12 and Figure 13 are polygonal maps of the landscape index and sample constraint factor *C*, respectively. LSI reflects the complexity of the overall landscape shape of the watershed units. The larger the value, the simpler the overall landscape shape. CONTAG describes the degree of agglomeration of patch types in the watershed units. The higher the value, the better the degree of agglomeration. SHEI describes the diversity of the patch distribution in the watershed units. The smaller the value, the simpler the patch type, and the smaller the diversity.

*N_S_* and *n* are obtained under the premise that the OA standard deviation *V*(${\bar{y}}_{st}$) equals 0.01. According to Figure 11, the value of *n* is often more than 1000, and most of them are distributed near 1750, whereas the values of *N_S_* are much smaller. This shows that the sample size *N_S_*, as obtained by the multi-level linear model considering spatial heterogeneity, can save more manpower and material resources in the accuracy assessment of a land cover dataset.

The green squares in Figure 11 indicate the relative size of the basin unit area, i.e., the scale difference. According to Figure 3b, there are evident scale differences among the basin units. In theory, the larger the scale of the watershed unit, the more samples are extracted, and the *n* values obtained based on the probability and statistics theory should show similar laws. However, the larger the scale of the unit, the smaller the *N_S_* values needed, such as the basin unit number (No.) 11, No. 20, and No. 35, and the smaller the unit, the larger the *N_S_* values, e.g., basin unit No. 14 and No. 17. According to Figure 12 and Figure 13, we can explain why *N_S_* changes with areas of the spatial unit. The values of the LSI, CONTAG, and SHEI in units No. 11, No. 20, and No. 35 are higher, indicating that these few units have a high patch aggregation, uniform patch distribution, and simple landscape shape, and thus weak spatial heterogeneity. Although the scale is large, the required sample size *N_S_* is small. However, the values of LSI and CONTAG in units No. 14 and No. 17 are low, and the SHEI values are high. They need a large sample size of *N_S_*. Therefore, by analysing the relationship between the sample size *N_S_* of the watershed units No. 11, No. 14, No. 17, No. 20, No. 35 and the landscape index, it is further demonstrated that the values calculated from the multi-level sample size estimation model (considering the spatial heterogeneity of land cover) are more reasonable than those calculated from the probability statistical model.

As noted above, *C* indicates how large of a sample is taken. If the land cover in different areas is homogeneous and the patch types and distribution are the same, the *C* values are similar. However, in Figure 12, the trend of *C* variation is similar to that of LSI, because the spatial distribution of the patches in different basin units is different. In an area with simple spatial distribution and weak spatial heterogeneity, *N_S_* is small, and the *C* value is high, i.e., *C* has a negative correlation to *N_S_*, whereas when *N_S_* is large and the *C* value is low, *C* has a positive correlation to *N_S_*.

Where the *C* values are the same, e.g., in basin units No. 16 and No. 17, because the LSI value of N_O_. 16 is high, the SHEI value is low, and the spatial heterogeneity of the land cover is weaker than that of No. 17. Because the two scales are similar, the sample size of No. 17 is larger. Although the C values in basin units No. 38 and No. 39 are the same, the LSI values of No. 38 are large, but the CONTAG and SHEI values are less than those of No. 39. Therefore, the difference in spatial heterogeneity between No. 38 and No. 39 is smaller than that between No. 16 and No. 17, resulting in a small sample size gap between No. 38 and No. 39. As a result, *C*, as a factor affecting the sample size of *N_S_*, is also influenced by the surface spatial heterogeneity.

## 5. Conclusions

The accuracy of a dataset is often the first problem to be considered in scientific research and field applications. Sample size calculation is the first step in performing an accuracy assessment of land cover products from satellite imagery. On the basis of a statistical model for the estimation of sample size, this study establishes a multi-level linear model for estimation of sample size by considering the scale effect and spatial heterogeneity. A watershed unit was introduced to obtain a spatial analysis unit, to avoid subjectivity in the selection of assessment units. Landscape indices were selected to indicate the spatial heterogeneity of the region.

The multi-level linear sample size estimation model shows that:

(1): All predictive variables can explain 79% of the variance of the sample size *N_S_*. The coefficients of the predictive variables of the model are significant, indicating that there is a strong relationship between the sample size *N_S_* and the independent variables. By comparing the sample size *N_S_* from the multi-level linear model with a sample size *n* based on probability and statistics theory, we see that the sample size of *N_S_* is much smaller than that of *n*. The smaller sample size can achieve the same performance as the statistical model and it contributes to the consideration of surface heterogeneity. The relative importance of the predicted variables in the model is calculated by using standardised regression coefficients and relative weights. The results show that the CONTAG and SHEI indicators (describing the diversity and dispersion of basin units, respectively) are relatively important, followed by LSI, sample constraint *C*, and sample size *n,* as calculated from probability sampling theory. According to the validation of the developed model, we can conclude that the smaller sample size from the developed estimation model can achieve the same performance as the statistical model while saving more time, cost, and energy in the accuracy assessment of land cover products.

(2): After performing three-fold cross-validation, the R^2^ value changes from 0.79 to 0.63. This means that the generalization of the sample size estimation model is still a problem, although the test of the model in the Anhui Province proved the validity of this estimation of sample size. Therefore, we need more work on the improvement and testing of the developed model for sample size estimation.

For a specific work on accuracy assessment, although the model established in this study cannot be directly applied, it is expected to provide an approach for the determination of sample size, by considering the study areas and the characteristics of the surface heterogeneity. In the future, we need to improve the developed model by applying this surface heterogeneity-concerned sample size estimation model to other study sites, aiming to assess the accuracy of a land cover dataset with as few samples as possible.

## Figures and Tables

**Table 1:** Land cover dataset reclassification and corresponding relation.

Globeland30		Land Use/Cover Change (LUCC)	
Index	Class Name	Index	Class Name
10	Cultivated Land	11	Paddy Land
		12	Dry Land
20	Forest	21	Forest
		23	Woods
		24	Others
30	Grassland	31	Dense Grass
		32	Moderate Grass
		33	Sparse Grass
40	Shrubland	22	Shrub
50	Wetland	46	Bottom Land
		64	Swampland
60	Water Bodies	41	Stream and Rivers
		42	Lakes
		43	Reservoir and Ponds
80	Artificial Surfaces	51	Urban Built-up
		52	Rural Settlements
		53	Others
90	Bare Land	65	Bare soil
		67	Others

**Table 2:** Parameter values from watershed unit Number 2.

	*W_h_*	*N_S_*																			
		50	100	150	200	250	300	350	400	450	500	550	600	650	700	750	800	850	900	950	1000
***S*_1_**	0.23	0.47	0.41	0.42	0.49	0.49	0.41	0.45	0.41	0.39	0.46	0.45	0.47	0.43	0.44	0.39	0.46	0.47	0.40	0.45	0.44
***S*_2_**	0.67	0.41	0.29	0.32	0.33	0.25	0.28	0.34	0.30	0.35	0.31	0.29	0.32	0.30	0.34	0.32	0.32	0.31	0.29	0.31	0.33
***S*_3_**	0.05	-	0.00	0.33	0.33	0.27	0.00	0.00	0.25	0.00	0.35	0.28	0.20	0.26	0.40	0.29	0.35	0.31	0.22	0.33	0.25
***S*_4_**	0.00	-	-	-	-	-	-	-	-	-	-	-	-	-	-	-	-	-	-	-	-
***S*_5_**	0.02	0.47	0.50	0.47	0.49	0.00	0.35	0.50	0.33	0.33	0.40	0.49	0.50	0.48	0.43	0.49	0.48	0.46	0.48	0.49	0.43
***S*_6_**	0.02	-	0.00	0.47	0.50	0.35	0.50	0.50	0.45	0.47	0.46	0.50	0.49	0.48	0.50	0.39	0.50	0.45	0.50	0.50	0.47
***S*_7_**	0.02	0.00	0.00	0.49	0.47	0.50	0.49	0.50	0.47	0.48	0.49	0.49	0.47	0.49	0.48	0.50	0.36	0.39	0.45	0.46	0.43
***S*_8_**	0.00	-	-	-	-	-	-	-	-	-	0.00	-	0.00	-	0.00	-	0.00	-	-	0.00	-

**Note:**$S_{1}$, …, $S_{8}$ corresponds to 10, 20, 30, 40, 50, 60, 80 and 90 land cover categories in Table 1 respectively, - represents that the stratum is not sampled in Globeland30.

**Table 3:** Landscape indicators used in this study.

Class	Name	Unit	Range
Area Metrics	Largest Patch Index (LPI)	%	(0,1)
Contrast Metrics	Mean Patch Size (MPS)	hm^2^	>0
Edge Metrics	Edge Density (ED)	m/hm^2^	--
	Patch density (PD)	--	>0
Shape Metrics	Landscape shape index (LSI)	--	≥1
	Area-weighted mean shape index (AWMSI)	--	(1,2)
	Area-weighted Mean patch fractal dimension (AWMPFD)	--	[1,2]
Proximity Metrics	Mean proximity index (MPI)	--	>0
Diversity Metrics	Shannon’s diversity index (SHDI)	--	≥0
	Patch richness density (PRD)	--	>0
	Shannon’s evenness index (SHEI)	--	(0,1)
Aggregation Metrics	Interspersion and Juxtaposition index (IJI)	%	(0,100)
	Contagion index (CONTAG)	%	(0,100)

**Table 4:** Multi-level regression coefficients of sample size *N_S_*.

	Estimate	Standard Error	*t* Value	Pr (>|t|)	Significance Codes
(Intercept)	−17,880	5846	−3.058	5.97 × 10^−3^	0.01
n	−0.278	0.076	−3.658	1.47 × 10^−3^	0.01
C	−9.392	1.344	−6.987	6.72 × 10^−7^	0.001
LSI	5.479	1.026	5.34	2.70 × 10^−5^	0.001
CONTAG	109.900	23.380	4.699	1.22 × 10^−4^	0.001
SHEI	7163	1465	4.89	7.78 × 10^−5^	0.001
AWMSI	−7.424	4.932	−1.505	1.47 × 10^−1^	1
AWMPFD	5803	3092	1.877	7.45 × 10^−2^	0.1
PRD	48,490	20,160	2.405	2.55 × 10^−2^	0.05
Residual standard error	95.28 on 21 degrees of freedom				
Multiple R^2^ squared	0.8384				
Adjusted R^2^ squared	0.7768				
F-statistic	13.62 on 8 and 21 DF				
*p*-value	<9.80 × 10^−7^				

**Table 5:** Modified multi-level regression coefficient of sample size *N_S_*.

	Estimate	Standard Error	*t* Value	Pr(>|t|)	Significance Codes
(Intercept)	−7159	1632	−4.386	1.98 × 10^−4^	0.001
n	−0.255	0.070	−3.627	1.34 × 10^−3^	0.01
C	−9.261	1.366	−6.781	5.16 × 10^−7^	0.001
LSI	4.210	0.762	5.523	1.11 × 10^−5^	0.001
CONTAG	77.900	16.960	4.592	1.17 × 10^−4^	0.001
SHEI	5085	960.500	5.294	1.98 × 10^−5^	0.001
Residual standard error	100.7 on 24 degrees of freedom				
Multiple R^2^ squared	0.7935				
Adjusted R^2^ squared	0.7505				
F-statistic	18.45 on 5 and 24 DF				
*p*-value	1.58 × 10^−7^				

**Table 6:** Cross-validation output results.

**Original R-square**	0.7935414
**Three-fold cross-validated R-square**	0.634445
**Change**	0.1590964

**Table 7:** Comparison of sample size and accuracy calculated from statistical model and this study (No. = Number).

	Method	Multi-LEVEL Model		Statistical Model		Absolute Value of OA Difference (%)	Sample Size Difference (N_S_-n)
Units Code		N_S_	OA (%)	n	OA (%)		
No.1_Ah		475	79.62	1511	79.56	0.06	1035
No.2_Ah		675	79.56	1388	79.54	0.02	714
No.3_Ah		1288	56.06	1561	56.63	0.57	274
No.4_Ah		1110	68.38	1620	68.40	0.02	510
No.5_Ah		485	77.11	1759	77.37	0.26	1274
No.6_Ah		956	75.10	1556	76.56	1.45	601
No.7_Ah		922	73.64	1591	69.94	3.71	669
No.8_Ah		879	66.78	2045	67.63	0.85	1166
No.9_Ah		978	69.19	1979	67.66	1.53	1002
No.10_Ah		1106	59.19	1902	59.94	0.75	796
No.11_Ah		696	52.65	2186	48.86	3.80	1490
No.12_Ah		1128	64.69	1384	66.16	1.47	256
No.13_Ah		912	61.95	2006	63.24	1.29	1093
No.14_Ah		790	63.29	1935	64.50	1.20	1145
		Sum = 12,399		Sum= 24,425		Average = 1.21	Sum = 12,026

**Table 8:** Standardised regression coefficients result.

Independent Variable	n	C	LSI	CONTAG	SHEI
Standardised regression coefficients	−3.60 × 10^−1^	−8.13 × 10^−1^	9.90 × 10^−1^	1.97	2.31
Relative importance level	5	4	3	2	1
